# The Relationship Between Atrial Fibrillation and the Systemic Immune Inflammation Index in Well-Controlled Hypertensive Patients with Newly Developed Atrial Fibrillation

**DOI:** 10.3390/jcm15072711

**Published:** 2026-04-03

**Authors:** Ümmü Taş, Sedat Taş, Erkan Alpaslan, Mehmet Eyüboğlu

**Affiliations:** Department of Cardiology, İzmir Demokrasi University, Göztepe, No:147, Buca, 35390 İzmir, Turkey; ummu.tas@gmail.com (Ü.T.); erkan8721@gmail.com (E.A.); mhmtybgl@gmail.com (M.E.)

**Keywords:** atrial fibrillation, chronic inflammation, hypertension, inflammatory marker, systemic immune-inflammation index

## Abstract

**Background:** Hypertension (HT) and atrial fibrillation (AF) are frequently coexisting conditions, with inflammation being a crucial pathophysiological mechanism shared by both. The systemic immune-inflammation index (SII), a newly introduced combined inflammatory marker, includes the parameters of platelets, neutrophils, and lymphocytes. Current literature lacks studies investigating the relationship between SII and newly developed AF in patients with controlled HT. Therefore, this study aimed to explore the association of SII with AF in hypertensive patients on anti-hypertensive therapy. **Methods:** This retrospective case–control study included 68 hypertensive patients with controlled blood pressure who underwent 24 h Holter monitoring. Patients were divided into two groups based on the presence of atrial fibrillation (AF-positive, n = 32; AF-negative, n = 36). SII values were calculated from complete blood counts. Demographic, clinical, and laboratory characteristics were compared between groups. Correlation analysis was performed to assess associations of SII with clinical parameters. Multivariable logistic regression identified independent predictors of AF. Optimal cut-off values for systemic immune-inflammation index and age were determined through receiver operating characteristic analysis. **Results:** Patients with AF were significantly older than those without AF (*p* < 0.01). The systemic immune-inflammation index was significantly higher in patients with atrial fibrillation (*p* = 0.02). Patients with AF also had higher heart rates (*p* = 0.04) and creatinine levels (*p* < 0.01). SII showed a moderate positive correlation with C-reactive protein (CRP) (r = 0.393, *p* < 0.01) and a weak correlation with heart rate (r = 0.251, *p* = 0.039). In multivariable analysis, SII (OR: 1.024, *p* = 0.04) and age (OR: 1.130, *p* < 0.01) was independently associated with AF. Receiver operating characteristic analysis determined an SII cut-off of 483.0 with a sensitivity of 53.1% and specificity of 52.8%. The age cut-off was found to be 63 years with sensitivity and specificity being 62.5% and 66.7%, respectively. **Conclusions:** The systemic immune-inflammation index was significantly elevated in newly diagnosed AF patients with well-controlled hypertension and was an independent predictor of AF. It is a simple, readily available biomarker that may assist in identifying hypertensive patients at high risk for the development of AF. These results should be validated in future studies, and the role of inflammation in the pathogenesis of atrial fibrillation should be further explored.

## 1. Introduction

Hypertension (HTN) is a major global health concern and a leading cause of cardiovascular morbidity and mortality. Recent experimental and clinical studies show that oxidative stress links HTN to two major pathways: inflammation and immune cell activation [1]. This indicates that immunological inflammation plays a pivotal role in the pathogenesis of HTN. Several studies have reported elevated levels of inflammatory markers such as C-reactive protein (CRP), interleukin 1 beta (IL-1β), IL-6, and tumor necrosis factor-alpha (TNF-α) in patients with HTN [2,3]. Histologically, HTN is characterized by increased smooth muscle and endothelial cell proliferation, medial hypertrophy, inflammation, and thrombosis. Patients with HTN remain at cardiovascular risk even after effectively controlling their blood pressure (BP). Possible underlying pathophysiological mechanisms include (i) endothelial dysfunction driven by inflammatory and immune signaling (e.g., TLRs, neutrophil-to-lymphocyte ratio (NLRP3) inflammasome), which leads to pro-thrombotic, pro-atherogenic changes independent of current BP [4]; and (ii) the persistent activation of immune cells in the blood vessel wall, including macrophages, neutrophils, and T cells, which continues even in controlled BP [5]. This indicates that the remaining cardiovascular risk stems from immune cell activation and persistent inflammation [6].

Atrial fibrillation (AF) is the most commonly observed arrhythmia in practice, which increases with age and affects approximately 14% of people [7]. The burden of AF results in serious health consequences such as stroke and heart failure; hence, early identification and treatment are warranted. Age, genetic predisposition, diabetes, HTN, and inflammatory markers all play a significant role in atrial structure and electrophysiological changes that predispose to AF [8,9]. AF typically progresses from sporadic, sudden episodes to more frequent and longer-lasting ones, eventually leading to chronic AF for most patients; higher AF rates are associated with worse outcomes in heart failure, ischemic stroke, and total mortality. Several recent studies highlight the critical role of inflammation in the pathogenesis of AF [10,11]. Atrial fibrosis is one of the key mechanisms through which AF develops; further increased inflammation leads not only to a greater incidence, but also longer duration and more persistent episodes of AF. Numerous inflammatory markers, including CRP, white blood cells, platelets, fibrinogen, TNF-α, interleukins, and NLR, have been associated with AF in studies conducted on different populations [12,13]. The systemic immune-inflammation index (SII), calculated from platelet, neutrophil, and lymphocyte counts, has recently emerged as a key prognostic marker in cardiovascular and other diseases. In patients with coronary artery disease (CAD), higher SII is linked to greater AF burden and increased likelihood of AF detection during long-term monitoring [14]. In heart failure, SII has been identified as an independent predictor of new-onset AF (NOAF) and adverse outcomes [15]. Furthermore, studies involving patients undergoing catheter ablation for AF have demonstrated that higher pre-procedural SII correlates with a higher risk of AF recurrence following the procedure [16]. Although several recent studies have reported an association between elevated SII and the presence or burden of AF in various clinical settings, as mentioned above [14,15,16,17], an important knowledge gap remains. Specifically, no study to date has examined the SII–AF relationship exclusively in patients with HTN well controlled with antihypertensive therapy. This population is clinically relevant because controlled HTN retains residual cardiovascular and arrhythmic risk that cannot be fully explained by hemodynamic factors alone, revealing a persistent inflammatory substrate. The heterogeneity of populations in previous studies—which frequently included patients with diabetes mellitus, CAD, heart failure, or uncontrolled BP—limits the generalizability of their findings to this specific group. Therefore, this study aimed to investigate the association between SII and newly detected AF in a well-defined cohort of HTN patients with controlled BP to isolate the inflammatory contribution to AF risk independently of comorbidities.

## 2. Material and Methods

**Figure 1:**
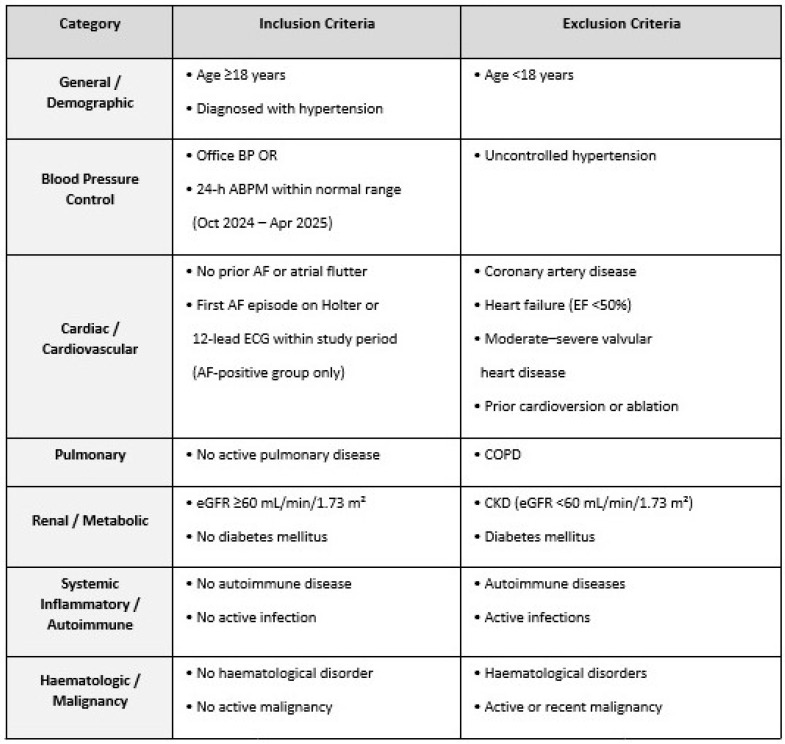
Summary of inclusion and exclusion criteria by conceptual category.

This retrospective case–control analysis was approved by the ethical committee of İzmir Demokrasi University, adhered to the principles established in the Declaration of Helsinki, and was conducted over a 7-month data collection period from 1 October 2023 to 30 April 2024. Hospital records were reviewed to identify patients with HTN whose BP was confirmed to be well controlled (defined as an office BP measurement of <140/90 mmHg or a 24-h ambulatory BP monitoring (Omron Healthcare, Kyoto, Japan) result within the normal range) documented within the study period. Newly developed (new-onset) AF was defined as the first documented episode of AF detected on 24 h Holter ECG monitoring (Risingmed Holter Monitor, Beijing, China) or on a 12-lead ECG recorded during the study period, without a history of AF or atrial flutter in the patient’s hospital medical records, antiarrhythmic therapy prescribed for AF, and cardioversion or ablation procedure for AF. The inclusion and exclusion criteria of the study are shown Figure 1.

In selected patients, SII was calculated using the following formula: platelet count × neutrophil count/lymphocyte count, derived from a single complete blood count (CBC) per patient. Blood samples were collected after a minimum 8 h overnight fast, in adherence to the routine morning admission blood collection protocol of our institution. For all patients, CBC was obtained during the routine admission blood work. All analyses were conducted in a single accredited central laboratory using a standardized automated hematology analyzer (Mindray Bio Medical Electronics Co., Ltd., Shenzhen, China). Participants were divided into two groups based on Holter ECG monitoring or 12-lead ECG results: Group 1, patients diagnosed with AF by Holter ECG monitoring or newly diagnosed during outpatient visits; and Group 2, patients without AF. A comparative analysis of both groups was performed using SII values and laboratory results.

The sample size was calculated a priori using G*Power V3.1.9.7 (two-independent-samples *t*-test, two-tailed). The primary endpoint for this calculation was the between-group difference in SII values (AF-positive vs. AF-negative). With a medium-conservative effect size (Cohen’s d = 0.65), a two-sided α of 0.05, and a power of 0.80, at least 60 participants were required (30 per group). Adding a 10% attrition allowance yielded a target sample of 66 participants. The final study enrolled 68 patients (AF+: n = 32; AF−: n = 36).

### Statistical Analysis

All statistical analyses were conducted with SPSS version 24.0. Categorical data are presented as raw numbers and percentages, whereas continuous data are presented as mean ± standard deviation and median. The Kolmogorov–Smirnov test was used to evaluate the normality of continuous variables. Between-group differences were assessed using the chi-square test for categorical data and the independent-sample *t*-test or Mann–Whitney U test for continuous data. Pearson’s correlation coefficient was used to analyze associations between SII and other continuous variables. Multivariable binary logistic regression—with age, SII, triglycerides, HDL cholesterol, and CRP as covariates—was performed to identify independent predictors of AF. Covariates for the multivariable model were selected a priori based on established clinical relevance as AF risk factors or metabolic confounders of the SII–AF relationship (age, CRP, TG, HDL), combined with SII as the primary exposure of interest. Age and SII were also significant in univariable analysis within the present dataset. Variables were selected based on clinical relevance, previously established associations with AF in the literature, and statistical significance in univariable analyses. Optimal cut-off values for SII and age were established by performing receiver operating characteristic (ROC) curve analysis. Statistical significance was defined as *p* < 0.05.

## 3. Results

The study enrolled 68 participants (88.2% female), comprising 32 (47.1%) AF-positive individuals based on 24-h Holter ECG or 12-lead ECG findings. The patients with AF were significantly older than those without AF (*p* < 0.001), and the SII values were significantly higher in the AF group (*p* = 0.020). Furthermore, the heart rate (*p* = 0.040) and creatinine levels (*p* = 0.010) were also higher in the AF group. Antihypertensive drug use was similarly distributed between groups (*p* = 0.667; Table 1). Detailed demographic, clinical, and laboratory characteristics are shown in Table 1.

SII was significantly and positively correlated with CRP (r = 0.393, *p* < 0.001) and heart rate (r = 0.251, *p* = 0.039) but not with other laboratory or clinical parameters (Table 2).

In multivariable analysis, SII and age were determined as factors increasing the risk of the outcome. SII was an independent predictor of AF (OR per 1-unit increase: 1.024, 95% CI: 1.001–1.047, *p* = 0.040; re-scaled OR per 10-unit increase: 1.27, 95% CI: 1.01–1.59), demonstrating that each 10-unit increment in SII was associated with a 27% increase in the odds of AF, emphasizing the role of systemic inflammation. Each additional year of age increased the odds by 13% (OR: 1.130, 95% CI: 1.062–1.172, *p* < 0.001). No significant relationships were observed for TG, HDL, and CRP (Table 3).

**Figure 2:**
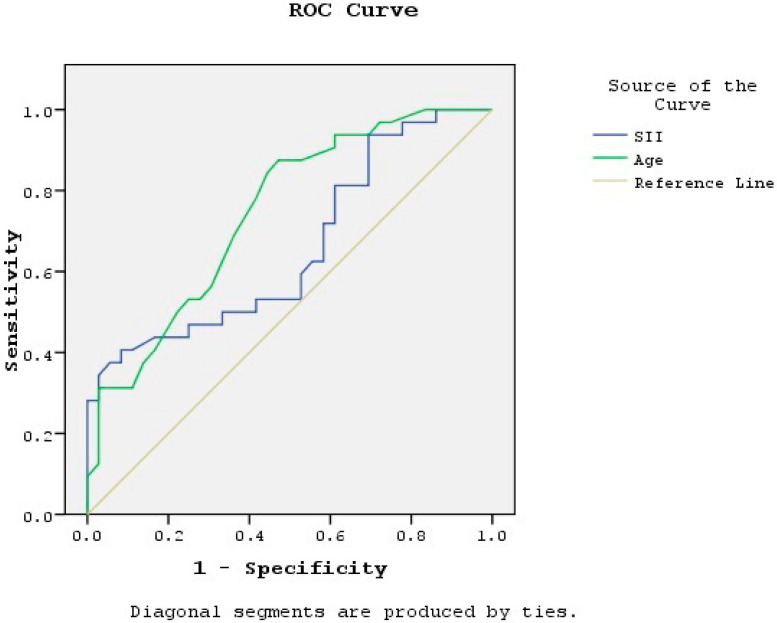
Receiver operating characteristic (ROC) curves for the systemic immune-inflammation index (SII) and age as predictors of newly developed atrial fibrillation in well-controlled hypertensive patients. SII: AUC = 0.659 (95% CI: 0.527–0.791; *p* = 0.025); optimal cut-off 483.0; sensitivity 53.1%, specificity 52.8%. Age: AUC = 0.744 (95% CI: 0.629–0.859; *p* = 0.001); optimal cut-off 63 years; sensitivity 62.5%, specificity 66.7%.

A ROC curve analysis was conducted to assess the discriminative ability of SII and age in predicting AF. For SII, the AUC was 0.659 (95% CI: 0.527–0.791; *p* = 0.025), with an optimal cut-off of 483.0 yielding a sensitivity of 53.1% and a specificity of 52.8%. For age, the AUC was 0.744 (95% CI: 0.629–0.859; *p* = 0.001), with an optimal cut-off of 63 years yielding a sensitivity of 62.5% and a specificity of 66.7% (Figure 2).

## 4. Discussion

The primary results were as follows. (i) Patients with newly diagnosed AF had significantly higher SII levels than those without AF in patients with HTN with well-controlled BP. (ii) Age and SII were identified as independent predictors of AF in multivariable analysis, which can be used separately for assessing the risk of developing AF. (iii) Significant correlations were observed between SII and CRP, as well as the heart rate, supporting the idea that SII could function as a marker for systemic inflammation and activation of the sympathetic nervous system. (iv) These findings add to an ever-growing list of evidence that supports inflammation’s key role in both the structural and electrical remodeling of atrial tissues, which finally results in the development of AF in patients with HTN with well-controlled BP.

Our findings are consistent with, but extend, the existing SII–AF evidence. Naser et al.’s study on 453 patients found a significant association between SII and AF; those with permanent AF had higher SII levels than those with paroxysmal AF, which is independently linked to AF load [18]. Zhao et al. reported that every standard deviation increase in log-SII was associated with a 150% increase in the incidence of new cases of paroxysmal AF; thus, a graded risk exists for developing more severe forms of this condition related to higher values for SII [19]. Ömür et al. confirmed a significant correlation between SII and CRP, which further supports the association between systemic inflammation and the severity of AF [20]. Collectively, these studies were conducted in populations with substantial comorbidity burdens—including established CAD, reduced ejection fraction, or post-ablation states. Conversely, this study demonstrates that the SII–AF association is detectable, even without these comorbidities, in a population where BP is pharmacologically controlled and baseline inflammatory activity is expected to be lower. This finding may indicate that the inflammatory substrate captured by SII is an independent contributor to AF risk rather than a secondary reflection of cardiac structural disease.

Multiple complex and interconnected mechanisms are involved in how high SII levels bring on arrhythmias in these patients. Recent studies demonstrate that inflammation plays a key role in AF by promoting structural remodeling and fibrosis within the atria, which increases the release of reactive oxygen species and induces tissue damage [11,13,15]. Elevated SII levels are consistent with a hypothesis in which persistent inflammation may help reduce the threshold for AF through pathways such as atrial fibrosis, oxidative stress, and autonomic instability; however, this cross-sectional study cannot establish causal directionality. Aune et al. argued that for both systolic and diastolic BP, the risk of AF increased even within the normal range of BP [21]. Zhang et al. confirmed that BP control alone may not significantly reduce the risk of AF recurrence in patients with a high NLR as an inflammation marker [22]. Even after BP normalization, immune cell activation—particularly neutrophil-mediated oxidative stress and T-cell-driven atrial fibrosis—persists in the atrial wall and drives structural remodeling independent of current hemodynamic load. Solak et al. speculated that HT is an autoimmune and inflammatory disease, not only a high BP situation [23]. Combined with the above-mentioned studies, autoimmunity and inflammation are speculated to be common in HTN and AF pathogenesis, and therefore, even if HTN is well controlled with antihypertensive agents, the inflammation persists and may cause AF.

The finding that SII was consistently significantly elevated in AF-positive patients despite similar antihypertensive treatment profiles across groups highlights an important mechanistic consideration: standard antihypertensive pharmacotherapy may not fully reverse the residual inflammatory state that predisposes patients with controlled HTN to AF. In this context, the nitric-oxide-soluble guanylate cyclase–cyclic GMP (NO–sGC–cGMP) pathway is specifically relevant: HTN-associated oxidative stress impairs sGC activity and reduces cGMP synthesis, thereby perpetuating atrial inflammation and fibrosis through pathways that standard antihypertensives do not directly target. In a recent study, novel agents that directly stimulate sGC have improved cardiovascular outcomes partly by restoring NO–sGC–cGMP signaling and reducing oxidative stress and endothelial dysfunction [24].

Recent studies from interventional electrophysiology further support the concept that inflammation is not merely a trigger for AF initiation but a persistent, modifiable determinant of its clinical trajectory. Mariani et al. found that the pulsed field ablation (PFA) procedure had significantly lower AF rates than high-power short-duration (HPSD) or very HPSD (vHPSD) radiofrequency ablation. Furthermore, they speculated that radiofrequency energy results in thermal-injury-driven inflammation (myocardial necrosis, pericardial reactions), whereas PFA (irreversible electroporation) elicits a more limited, non-thermal inflammatory response with preserved tissue selectivity [25]. Matteucci et al. confirmed that inflammatory responses triggered during AF treatment affect not only electrophysiological outcomes, but also patient-centered outcomes, including symptom burden and quality of life. They also indicated that less-invasive energy profiles (PFA) are associated with a more favorable symptom recovery trajectory [26]. Collectively, these findings support a framework in which systemic inflammation, as captured by accessible indices such as SII, is relevant not only to the risk of AF initiation—as demonstrated in this study—but also to the atrial substrate’s vulnerability to remodeling, its response to therapeutic intervention, and the patient’s symptomatic recovery.

The strong relationship between SII and heart rate denotes that sympathetic activity can trigger AF. In addition, age is independently associated with AF, underscoring the relationship between myocardial aging and inflammation-induced atrial vulnerability. Traditional metabolic markers such as cholesterol levels, glucose, and HbA1c did not exhibit significant differences between groups, nor did they correlate with SII, thereby indicating that inflammation may play a more dominant role than metabolic dysregulation in the early pathogenesis of AF among patients with HTN. The cumulative effect of these changes significantly decreases the threshold for triggering AF, making people more prone to arrhythmias even when standard risk factors such as BP, lipids, and glucose are well controlled, as in our study.

This study underscores this relationship by demonstrating that even among patients with HTN with controlled BP—who would generally have lower baseline inflammatory activity—SII can still be considered an important marker for evaluating the risk for developing AF in that population. A recent review aligned with our study, which evaluated how chronic low-grade inflammation affects the structural and electrical remodeling of the atria as well as its role in new AF cases and recurrences [27]. Furthermore, age was found to be a more significant independent predictor of new AF cases. This finding agrees with those of earlier studies that found a sharp increase in such incidences with increasing age [28]. The relationship between age and SII brings out the effects of biological aging plus inflamed states on remodeling within the atrium.

In contrast to previous studies that assessed cohorts with uncontrolled HTN [29], this study examined subjects with HTN with well-controlled BP and without any comorbidities. This design minimizes confounding variables associated with uncontrolled HTN and comorbidities, thus allowing a more direct assessment of inflammatory mechanisms. This study was limited to early or first-detected AF to differentiate it from that of previous studies that largely focused on persistent AF; thus, inflammatory changes could be detected that might be associated with the initiation of arrhythmia rather than the consequences of chronic structural remodeling. In this context, early inflammatory changes are somehow critically involved in the pathogenesis of AF. Increased inflammatory activity may be an epiphenomenon reflecting vascular aging, arterial stiffness, and autonomic dysfunction, which all predispose patients to an increased risk of AF. Consequently, although acute inflammatory changes might provide clues regarding the mechanism that induces AF, one must determine whether these changes are actually causative or merely indicative of more widespread pathological processes. Further longitudinal studies will be needed to completely understand these relationships.

To our knowledge, this is among the first studies to assess the association between NOAF and SII among patients with HTN with well-controlled BP. The study results demonstrate that SII can be used as a biomarker for identifying patients with HTN who are at an increased risk of AF, even if their BP measurements are within normal limits. High SII values should be detected early to allow appropriate rhythm monitoring and treatment, which will, in turn, help reduce AF-related complications such as stroke or heart failure. This particular population helps define the association between inflammation-related risk and the confounding effects of uncontrolled BP; hence, this group should be closely monitored. Timely interventions can reduce the negative effects of AF on these patients’ lives and improve outcomes for them. Further studies should clarify how SII plays a role in the onset of AF, as well as create a standardized protocol.

This study is limited due to its retrospective and cross-sectional design, which does not allow the establishment of causal relationships between SII and AF. However, whether elevated SII actually plays a direct role in the pathogenesis of AF or whether it simply reflects a secondary inflammatory response remains unknown. Second, data from one center with the exclusion of comorbidities do not adequately represent the general population, thus limiting generalizability. Third, the small sample size limits the statistical power and may not accurately depict variability in the parameters studied. Fourth, smoking was found not to be statistically significant for risk factors of AF, contrary to previous studies, but it may still apply to some individuals. Fifth, the lack of echocardiography data and atrial size indices precluded correlation between inflammation and key atrial structural features associated with susceptibility for AF. Sixth, inflammatory markers other than CRP have not been evaluated—IL-6, TNF-α, and fibrinogen—thus preventing direct mechanistic comparisons between SII and other known inflammatory biomarkers. Seventh, the multivariable logistic regression model included five covariates against 32 outcome events, yielding an events-per-variable ratio of 6.4—below the recommended threshold of 10. Overfitting cannot therefore be excluded, and the results should be considered hypothesis-generating, pending confirmation in larger prospective cohorts. Eighth, the cohort was heavily female-predominant (88.2% women), which substantially limits generalizability to male patients with HTN, and, finally, the lack of long-term follow-up data prevents us from evaluating the association between baseline SII levels and clinical outcomes, such as stroke or AF progression.

## 5. Conclusions

The SII was significantly higher in patients with HTN with newly detected AF compared to those without AF, irrespective of well-controlled BP in all study participants and the absence of confounding comorbidities. These data indicate an association between increased systemic inflammation, as represented by SII, and the presence of newly detected AF in patients with well-controlled HTN; whether this reflects a causal contribution or a coincident inflammatory state cannot be determined from this cross-sectional study.

## Figures and Tables

**Table 1:** Comparison of clinical and laboratory parameters between patients with and without atrial fibrillation.

Variable	AF Present (Mean)	AF Absent (Mean)	t-Statistic	*p*-Value
Age (years)	67.7 ± 9.9	57.8 ± 10.8	4.307	**<0.001**
Gender (male/female)	5/27	3/33		0.350
Smoking	0	2		0.170
SII	666.8 ± 475.5	454.1 ± 184.3	2.133	**0.020**
CRP (mg/L)	0.69 ± 0.64	0.58 ± 0.51	0.599	0.480
AST (U/L)	15.9 ± 5.3	16.6 ± 7.7	−0.149	0.660
ALT (U/L)	18.2 ± 5.9	16.7 ± 4.1	1.474	0.220
Total Cholesterol (mg/dL)	208.6 ± 41.5	219.0 ± 35.0	−0.834	0.270
LDL (mg/dL)	127.3 ± 36.6	134.6 ± 37.0	−0.434	0.410
HDL (mg/dL)	55.5 ± 9.8	54.5 ± 13.5	0.864	0.730
Triglycerides (mg/dL)	129.5 ± 37.8	148.3 ± 103.6	−1.623	0.330
Glucose (mg/dL)	96.9 ± 12.0	100.7 ± 21.3	−0.814	0.360
HbA1c (%)	5.8 ± 0.3	5.7 ± 0.3	0.809	0.500
Creatinine (mg/dL)	0.79 ± 0.16	0.67 ± 0.11	2.388	**<0.001**
TSH (µIU/mL)	2.59 ± 2.02	2.22 ± 1.44	0.991	0.370
Heart Rate (bpm)	85.4 ± 22.5	76.7 ± 10.9	2.116	**0.040**
Medication (n, %)				0.660
ARB + CCB (n, %)	5 (15.6)	4 (11.1)		
ACEİ + CCB (n, %)	4 (12.5)	6 (16.7)		
CCB + BB (n, %)	0 (0.0%)	1 (2.8%)		
ARB (n, %)	4 (12.5)	4 (11.1)		
ACEI (n, %)	3 (9.4)	2 (5.6)		
DIU (n, %)	1 (3.1)	1 (2.8)		
ARB + BB (n, %)	5 (15.6)	2 (5.6)		
BB (n, %)	4 (12.5)	5 (13.9)		
ACEI + BB (n, %)	1 (3.1)	0 (0.0)		
BB + DIU (n, %)	2 (6.3)	1 (2.8)		
ARB + DIU (n, %)	3 (9.4)	8 (22.2)		
ACEI + DIU (n, %)	0 (0.0)	2 (5.6)		

AF: Atrial fibrillation; SII: Systemic immune-inflammation index; CRP: C-reactive protein; AST: Aspartate aminotransferase; ALT: Alanine aminotransferase; LDL: Low-density lipoprotein; HDL: High-density lipoprotein; TSH: Thyroid-stimulating hormone; HbA1c: Hemoglobin A1c; ACEI: Angiotensin-converting enzyme inhibitor; ARB: Angiotensin II receptor blocker; BB: Beta-blocker; CCB: Calcium channel blocker; DIU: Diuretic. Bold values indicate statistical significance (*p* < 0.05).

**Table 2:** Correlation of SII with clinical and laboratory variables.

SII	Pearson r	*p*-Value
Age	0.064	0.606
CRP	0.393	**<0.001**
AST	0.035	0.774
ALT	0.138	0.260
Total Cholesterol	−0.078	0.529
LDL	−0.077	0.533
HDL	−0.075	0.541
Triglycerides	0.057	0.644
Fasting Glucose	0.082	0.505
HbA1c	0.077	0.530
CRE	−0.115	0.351
TSH	−0.116	0.345
Heart Rate (HR)	0.251	**0.039**

SII: Systemic immune-inflammation index; CRP: C-reactive protein; AST: Aspartate aminotransferase; ALT: Alanine aminotransferase; T-COL: Total cholesterol; LDL: Low-density lipoprotein; HDL: High-density lipoprotein; TG: Triglycerides; FG: Fasting glucose; HbA1c: Hemoglobin A1c; CRE: Creatinine; TSH: Thyroid-stimulating hormone; HR: Heart rate. Bold values indicate statistical significance (*p* < 0.05).

**Table 3:** Multivariable logistic regression analysis of independent predictors for atrial fibrillation.

Independent Variables	OR (95% CI)	*p*
SII (Per 10-Unit)	1.270 (1.010–1.600)	**0.04**
Age	1.130 (1.062–1.172)	**<0.01**
TG	1.003 (0.992–1.015)	0.53
HDL	0.998 (0.947–1.052)	0.95
CRP	0.819 (0.358–2.256)	0.81

SII: Systemic immune-inflammation index, TG: Triglyceride, HDL: High-density lipoprotein, CRP: C-reactive protein. Bold values indicate statistical significance (*p* < 0.05).

## Data Availability

The data presented in this study are available on request from the corresponding author. (The data are not publicly available due to privacy and ethical restrictions, as per the decision of the İzmir Demokrasi University Ethics Committee.)

## Abbreviations

AF	Atrial fibrillation
BP	Blood pressure
CAD	Coronary artery disease
CBC	Complete blood count
CRP	C-reactive protein
PFA	Pulsed field ablation
ROC	Receiver operating characteristic
SII	Systemic immune-inflammation index
